# A minireview on the utilization of petroleum coke as a precursor for carbon-based nanomaterials (CNMs): perspectives and potential applications

**DOI:** 10.1039/d4ra01196a

**Published:** 2024-06-20

**Authors:** Rivaldo Leonn Bezerra Cabral, Edney Rafael Viana Pinheiro Galvão, Pierre Basílio Almeida Fechine, Felipe Mendonça Fontes Galvão, José Heriberto Oliveira do Nascimento

**Affiliations:** a Postgraduate Program in Chemical Engineering, Center of Technology, Federal University of Rio Grande do Norte CEP 59072970 Natal RN Brazil systemriva38@gmail.com Heriberto.nascimento@ufrn.br; b Departament of Petroleum Engineering, Federal University of Rio Grande do Norte CEP 59078900 Natal RN Brazil; c Postgraduate Program in Textile Engineering, Center of Technology, Federal University of Rio Grande do Norte Natal RN Brazil; d Advanced Materials Chemistry Group (GQMat), Department of Analytical Chemistry and Physical Chemistry, Federal University of Ceará – UFC Campus do Pici, CP 12100 CEP 60451-970 Fortaleza CE Brazil

## Abstract

The remarkable properties of carbon-based nanomaterials (CNMs) have stimulated a significant increase in studies on different 0D, 1D and 2D nanostructures, which have promising applications in various fields of science and technology. However, the use of graphite as a raw material, which is essential for their production, limits the scalability of these nanostructures. In this context, petroleum coke (PC), a by-product of the coking process in petrochemical industry with a high carbon content (>80 wt%), is emerging as an attractive and low-cost option for the synthesis of carbonaceous nanostructures. This brief review presents recent research related to the use of PC as a precursor for CNMs, such as graphene and its oxidized (GO) and reduced (RGO) variants, among other carbon-based nanostructures. The work highlights the performance of these materials in specific areas of application. In addition, this review describes and analyzes strategies for transforming low-cost, environmentally friendly waste into advanced technological innovations with greater added value, in line with the UN's 2030 Agenda.

## Abstract 2

A mini-review on the latest research on the synthesis and applications of carbon-based nanomaterials obtained from petroleum coke and their implications as a by-product for the manufacture of materials in the global market.

## Graphical abstract

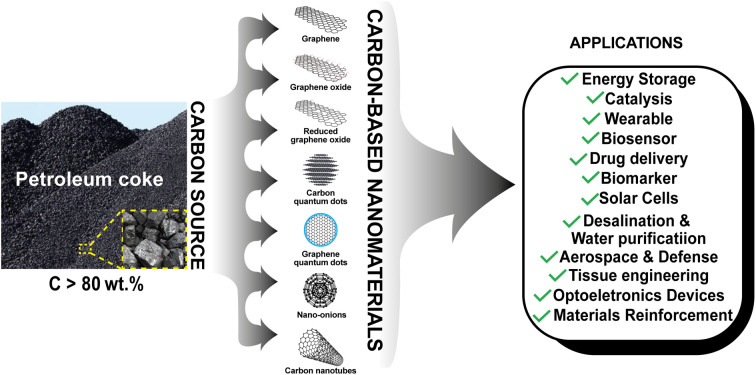

## Introduction

**Fig. 1:**
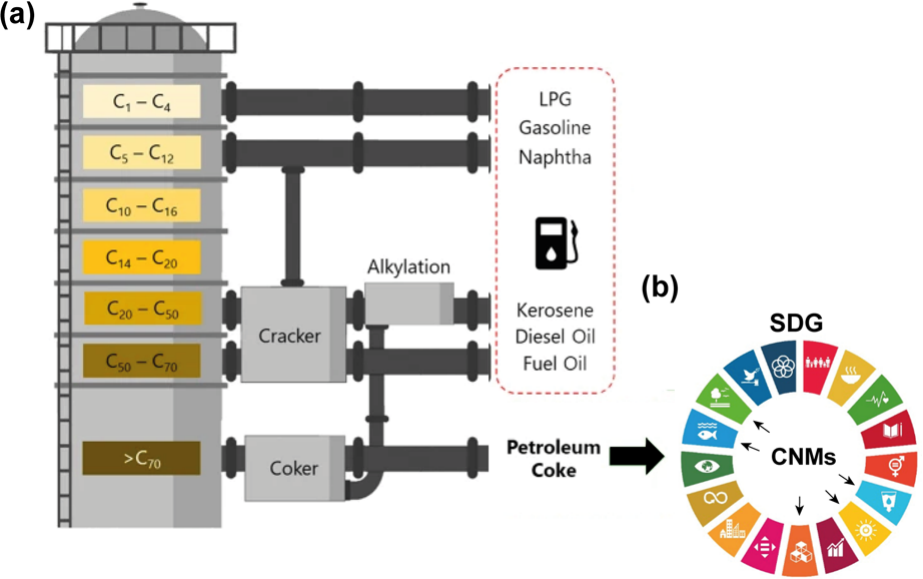
(a) Simplified scheme of oil refining operations and products, including petcoke. (b) Obtaining CNMs from petroleum coke as a potential solution for the 2030 Agenda for sustainable development, mainly involving (6) clean water and sanitation, (7) affordable and clean energy, (9) industry, innovation, and infrastructure, (14) life below water, and (15) life on land. Reproduced (adapted) with permission from ref. 10. Copyright© 2021, npj 2D Materials and Applications.

**Fig. 2:**
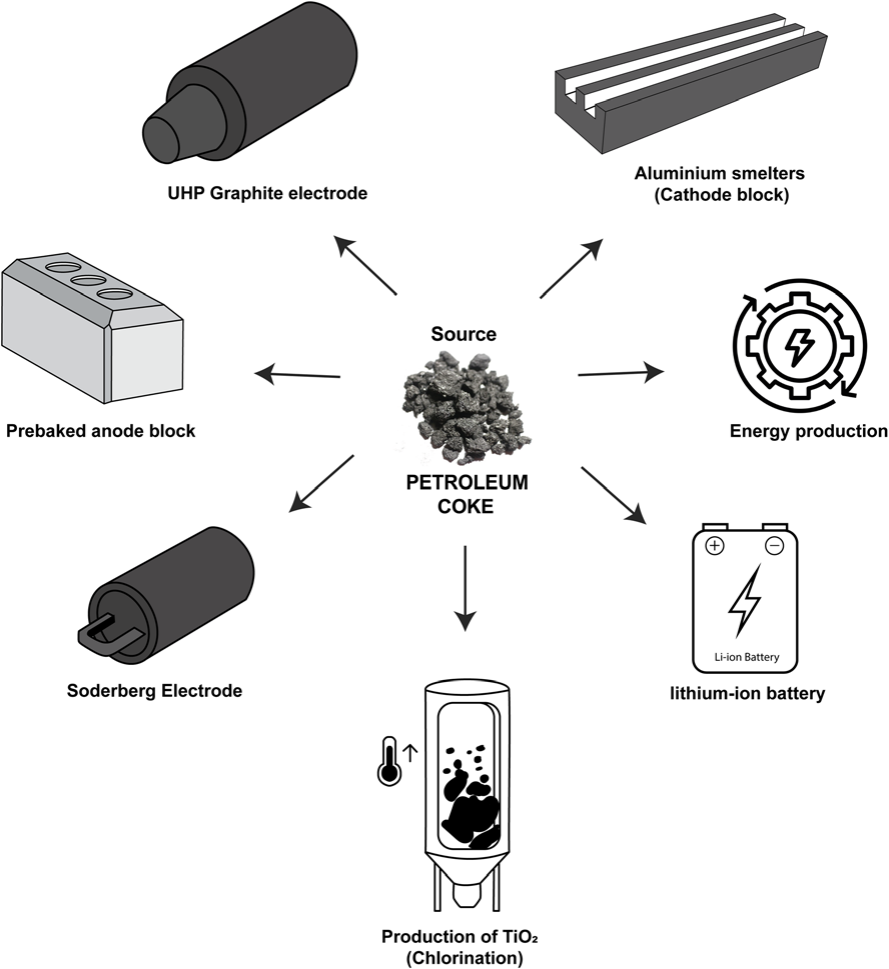
Different applications of petroleum coke as a carbon source.

In oil refineries, the gases released from the heating of crude oil are condensed and separated through different channels that lead to the formation of products such as gasoline, diesel, kerosene, naphtha, and lubricants (Fig. 1a).^1,2^ In addition to these petroleum products, the delayed coking process generates a dark granular solid byproduct called petroleum coke, petcoke, green coke, or green petroleum coke (GPC). Coking is a thermal cracking process without the use of catalysts, where an endothermic reaction takes place in heavy hydrogen-deficient tailings, such as asphaltenes and resins, forming coke. This byproduct is characterized by its high calorific value, low ash content, low cost compared to coal, wide availability, and high carbon content (>80 wt%).^3,4^ Consequently, this material is used as a precursor for energy generation,^5^ steel recycling in the electric arc furnace (UHP graphite electrode), graphite anode manufacturing in the aluminum smelting industry (prebaked carbon anode and aluminum smelters),^6,7^ titanium oxide production (chlorination),^8^ activated carbon synthesis,^9^ and anode materials for lithium-ion batteries,^3^ as shown in Fig. 2.

However, the increase in coking capacity has raised concerns about the potential impacts of petroleum coke use, which can have harmful effects on the health of living organisms and the environment.^11,12^ These harmful effects occur mainly through its combustion, emitting greenhouse gases, which contribute to global warming,^13^ including the sulfur dioxide (SO_2_) present in acid rain, making it harmful to animals and plants.^14^ Given these adverse effects, the petrochemical industry encounters a major challenge of managing this product in a sustainable way, principally when it comes to the Sustainable Development Goals (SDG) of the 2030 Agenda adopted by the UN member countries, which have the premise of enhancing the world's development and improving the quality of life of all people.^15^ Companies should be concerned with producing any material or creating products that are functional, economical, and safe, thus in line with the seventeen SDGs of the 2030 Agenda. Thus, nanotechnology appears to have tools that enable the growth of new green materials and processes, while being aligned with these policies (Fig. 1b).^16^ Therefore, there is much effort to redirect the by-product streams from oil refining to other ways that provide greater, sustainable, safe, and value-added materials.

In this sense, the development of carbon-based nanomaterials (CNMs) products using petroleum coke as a raw material is a reality, considering that a rich source of carbon can become a necessary resource as an abundant low-cost input for nanoproducts manufacturing. As a result, this by-product acquires a prominent role compared to other profitable products in the petrochemical market and other industrial segments. In this minireview, we describe recent developments for obtaining major carbon-based nanomaterials from petroleum coke. The chemical aspects of the synthesis and its market potential as a precursor for nanomaterials manufacturing are highlighted, including advances cited to better understand the context.

## Petroleum coke

The process of petroleum coke formation is based on thermally breaking the side chains of polycyclic aromatic hydrocarbons (PAHs) that make up the heavy feed in oil distillation columns,^17^ through fluid coking and delayed coking methods. In the delayed coking process, vacuum distillation residues or heavy distillates remain for short periods in horizontal ovens, where an endothermic reaction takes place that heats to temperatures of 450–510 °C at low pressures (0–300 kPa) until thermal cracking. Coking is “delayed” until the material is fed into cylindrical containers near the heater in which it is cracked, producing solid coke and gases, while the coke is cut in another container. In the fluid coking process, the raw material is sprayed onto a fluidized bed of hot coke particles which react on its surface, then crack, producing coke as thin layers which are deposited on the coke itself.^18^

Not only is delayed coking apparently a simpler method, it is also more widely used in refineries due to its lower capital cost and higher yield of coke produced than fluid coking.^19^ The United States has a coking capacity of 2.7 Mbbl per day of coke through delayed coking, approximately eighteen times greater than the fluid coking capacity of around 16 Kbbl per day.^20^ The coke yield from fluid coking is approximately 68% of the coke yield from delayed coking.^21^ However, the selection of the coking method will determine the purpose for which the crude oil coke will be used. Depending on the amount of fossil material, refineries produce approximately 75 million tons per year of green petroleum coke.^22^

**Fig. 3:**
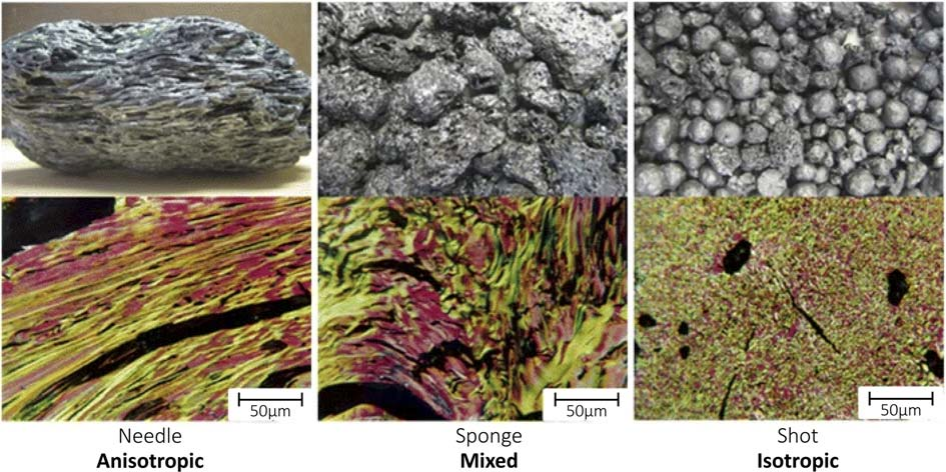
Petroleum coke types and optical textures. Reproduced (adapted) with permission from ref. 24. Copyright© 2015, The Minerals, Metals & Materials Society.

The process of delayed coking of crude oil can produce different types of petroleum coke, which depend on the temperature, time process and quality of raw material.^23^ Among the main petcoke types obtained are sponge coke, needle coke and shot coke (Fig. 3). Sponge coke is the most common petroleum coke type, which has an opaque black color and amorphous appearance. Visibly, it is easily identifiable by its porosity, which gives it a texture like the texture of a sponge, and it is produced from vacuum distillation residues with moderate concentration of PAHs that constitute the asphaltenes. Thus, it has low added value and is used as a solid fuel.^19^

Needle coke (NC) is a high-quality petroleum coke that also serves as a raw material for the production of artificial graphite, whose formation by thermal cracking of aromatic fractions produces graphite crystals of “needle shaped coke” structures oriented in a single direction. Because it has a low content of ash and sulfur, high crystallinity, high thermal and electrical conductivity and low coefficient of thermal expansion (CTE < 2.0 × 10^−6^ K^−1^),^24^ NC has attracted a lot of attention as an anode material, mainly to produce graphite electrodes applied in electric arc furnaces and for potassium-ion batteries (PIBs),^25^ so it is commercially more valued.^26^ However, studies have been conducted on the storage mechanism functional and structural evolution of the solid-electrolyte interface (SEI) in ester- and ether-based electrolytes.

Shot coke is another petcoke type that has a mix of shapes ranging from agglomerated blocks to more defined forms, such as individual spheres, and agglomerated spheres of different sizes, where it is produced from heavy oil feedstock^27^ with a high content of resins and asphaltenes containing high molecular weight, resulting in a material with CTE typically in the range of 3.5–4.8 × 10^−6^ K^−1^, and is the preferred structure for anode production.^24^

Furthermore, the physical and chemical characteristics of petroleum coke will determine the specific purpose for which it will be used, although petroleum coke is substantially made from carbon.^28^ For example, a higher concentration of heavy metals and sulfur in petroleum coke^4^ is more intended for fuel in power generation or cement manufacturing.^29^ Otherwise, a valorization of this product is employed due to moisture and hydrocarbon content reduction through by calcination process of petroleum coke used in the anode manufacturing for aluminum production, and for graphite electrodes in the electric arc furnaces.^30^ However, the large-scale production of CNMs from oil refining by-products is still incipient.

Although the characteristics of petroleum coke vary and depend not only on the crude oil source, but also on the quality specifications dictated by refineries, petroleum coke can be characterized according to its graphitization capacity for amorphous carbon materials of the soft carbon or hard carbon type. In most cases, HCs are ungraphitized forms of carbon materials, whose structure has a high degree of disordering and greater spacing between layers,^31^ such as polyaniline, epoxy resin, biomass and synthetic resin, *etc.* Even though HC cannot be graphitized even at 3000 °C,^32^ it is a potential anode candidate for sodium ion batteries (SIB) due to its high specific surface area, high electrical conductivity, abundance of resources and low cost.^33^ In terms of electrochemical performance, HC is the first active anode material to achieve productivity in SIB. In addition, HCs are attractive due to their abundance, cost-effectiveness, and the fact that the material can be obtained from biowaste or biological resources. However, numerous disadvantages, including its low initial coulombic efficiency and voltage hysteresis, limit their use in SIB.^34^

Unlike HC, soft carbons are carbon materials that can be completely graphitized at 3000 °C, such as pitch and coal tar pitch precursors.^31^ Specifically, soft carbon is composed of short-range sp^2^ type carbon clusters in a long-range disordered amorphous texture. This special microstructure is advantageous because it improves the migration of electrons and K^+^, contributing to an adequate rate and stability of the cycle. It has therefore emerged as one of the most promising anode materials compared to graphite and hard carbon.^34^

CNMs have a range of remarkable characteristics in several industrial sectors,^35^ among them are carbon allotropes: graphite, diamond, graphene, oxidized (GO) and reduced (rGO) graphene, carbon nanotubes (CNTs), fullerenes, carbon (CQDs) and graphene (GQDs) quantum dots, and others. In this group of carbonaceous nanomaterials, graphene has attracted increasing interest in research in the most diverse scientific areas because of its excellent physicochemical, thermal, mechanical and electrical properties, which make graphene one of the most studied CNMs.^36–43^ However, obtaining it in a cheaper and industrially scalable way is still a challenge. Thus, using other more abundant lower cost resources, such as petcoke, can be a great alternative.

The use of petroleum coke as a precursor for the synthesis of different CNMs is directly related to the fact that it is an abundant and low-cost reserve with high carbon content.^44^ In addition, compared to other carbon-based by-products, such as coal tar, petroleum coke has a higher PAH content.^45^ PAHs are important molecules for the formation of graphene-like nanostructures, as well as acting directly on the photoluminescent properties of CQD^46^ and graphene.^47^ As soon as the raw material goes through the calcination process at high temperatures (*T* > 1200 °C),^24^ dehydrogenation and some desulphurization reactions take place, removing moisture and volatile materials, and reducing impurities such as sulphur, nitrogen and other heteroatoms, as shown in Table 1. This results in a precursor with larger graphite regions and higher density, which are responsible for obtaining different CNMs.

**Table 1:** Typical specifications of different GPC and CPC^48^

Properties	Typical specification	
	GPC	CPC
Moisture, wt%	8–12	<0.3
Volatile matter, wt%	<12	<0.5
Ash, wt%	<0.4	0.1
Sulfur, wt%	3.5–7.5	<0.5
Nitrogen, ppmw	6000	<50
Nickel, ppmw	<500	<7

### Classification of nanomaterials

**Fig. 4:**
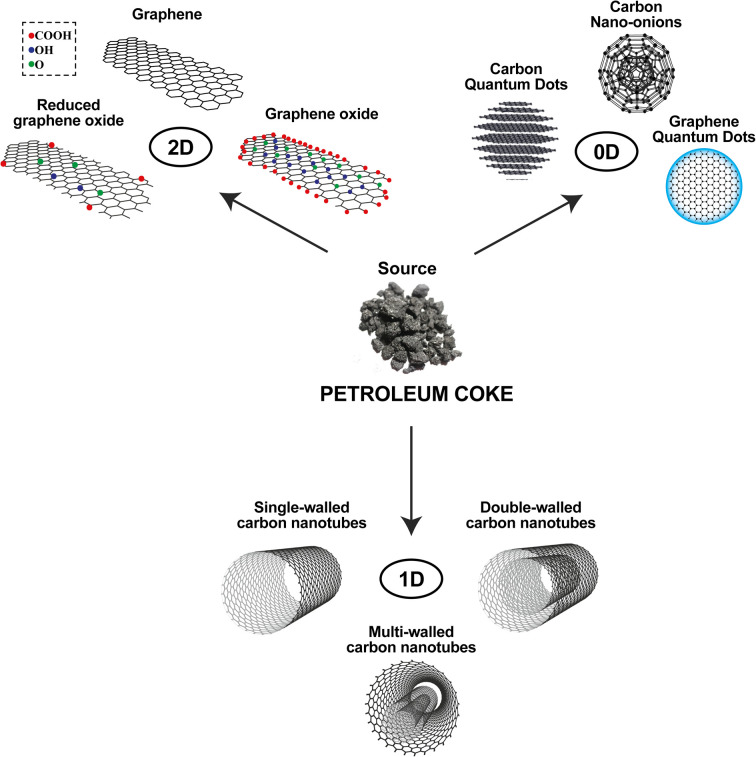
Classification of nanomaterials obtained from petroleum coke on the basis of dimensionality.

Nanomaterials are classified according to the predominance of the nanoscale as a function of dimension (between 1 and 100 nm) of the nanostructure, as shown in Fig. 4. One-dimensional (1D) CNMs are characterized by nanostructures with a single dimension of <100 nm; two-dimensional (2D) CNMs are nanostructures with two dimensions of <100 nm; zero-dimensional (0D) CNMs are nanostructures with no dimension of >100 nm. Although there are three-dimensional (3D) CNMs whose nanostructures are not confined to any dimension on the nanoscale,^49^ such as graphite and diamonds, there are no reports of direct applications of CNMs synthesized from petroleum coke.

### Graphene

Graphene is a nanomaterial characterized by being a unique two-dimensional sheet of structurally distributed carbon atoms packed in a lattice hexagonal. This type of carbonaceous material consists of an extremely thin layer graphite. For this reason, its nomenclature is composed of the prefix “graph” (derived from graphite) and the suffix “ene” (derived from PAHs such as naphthalene, anthracene, *etc.*). Because it is an isolated one-layer nanomaterial of aromatic structure with sp^2^ hybridizations,^36^ graphene has unique properties that are different from those of carbon materials, such as a high surface area,^37^ high transmittance,^38^ a fracture strength that is about 200 times higher than that of structural steel (130 GPa), high Young's modulus value (∼1 TPa),^39^ thermal conductivity that is five times higher than that of diamond (∼5000 W (m^−1^ K^−1^)),^40^ high charge carrier mobility (∼200 000 cm^2^ (V^−1^ S^−1^)),^41^ complete impermeability to any gases,^42,50^ and hydrophobicity.^43^ These and other competencies of the material indicate the importance of graphene in the technological development of new products for different applications.

There are several methods for obtaining graphene. The Hummers' method is the best known method,^51^ which consists of graphite oxidation by acid treatment and subsequent reduction (chemical or thermal). Other techniques to obtain graphene and its derivatives are performed under top-down (mechanical exfoliation,^52^ electrochemical exfoliation (ECE),^53^ ball milling,^54^ acid dissolution,^55^ and lithography^56^) and bottom-up (electrochemical reduction,^57^ sonochemical,^58^ microwave irradiation,^59^ hydrothermal,^60^ chemical vapor deposition,^61^ physical vapor deposition,^62^ arc discharge,^63^ solvothermal and sonication^64^ and green synthesis^65^) approaches. However, the process yield does not generate enough nanomaterial. Furthermore, it involves a high production time and the use of strong oxidizing agents that limit its large-scale fabrication.^66^

Graphite is still the most widely used precursor for graphene synthesis because it comprises its basal structural unit,^67^ due to the single-layer thickness of carbon. However, graphite is a finite source, where the largest portion of this mineral is unusable for graphene production because it is structurally composed of amorphous regions, as well as containing silicate minerals and minerals, meaning that only 10% to 15% of graphite is high purity graphitic carbon.^10^ For this reason, considerable effort has gone into producing graphene from other sources that have high carbon content in their composition, such as petroleum coke.

**Fig. 5:**
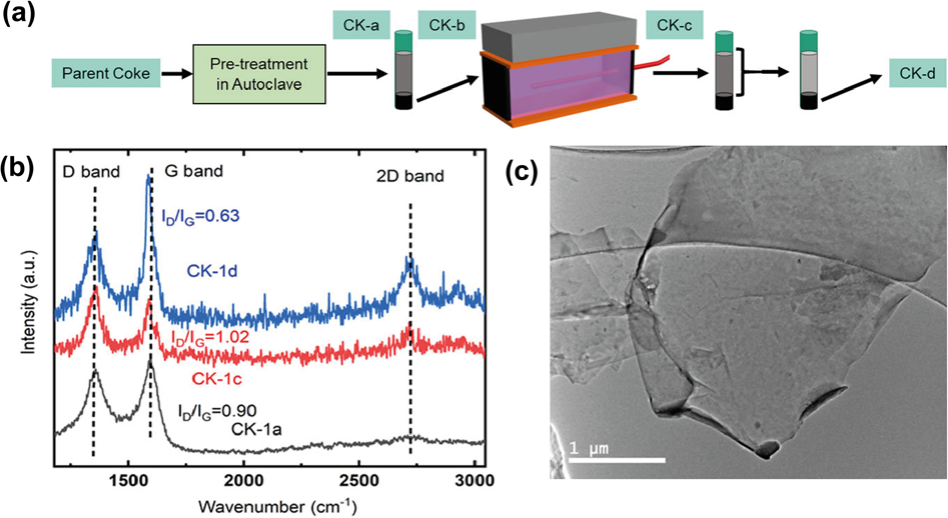
(a) Graphene synthesis process *via* electrochemical exfoliation (ECE) from petroleum coke (CK-a: washing and pretreatment, CK-b: separation by pre-reaction centrifugation, CK-c: ECE process, and CK-d: separation by post-reaction centrifugation), (b) D, G and 2D bands from the Raman spectra of CK-1a (raw material), CK-1c (material after ECE process), and CK-1d (material after post-reaction centrifugation process) with the respective *I*_D_/*I*_G_ ratios. (c) TEM images of ECE graphene. Reproduced (adapted) with permission from ref. 10. Copyright© 2021, npj 2D Materials and Applications.

For example, Saha *et al.* (2021) environmentally and economically sustainable synthesized graphene nanosheets by electrochemical exfoliation (ECE) using needle coke,^10^ as shown in Fig. 5a. The collaborators identified the 2D peak presence in the Raman spectra of the centrifuged nanomaterial after the electrochemical exfoliation reaction, in contrast to the original coke where the 2D peak is absent (Fig. 5b). Raman spectra provide valuable information about carbonaceous materials. Although the yield of graphene produced is still lower than typical yields obtained from the electrochemical exfoliation of graphite,^68,69^ the Raman spectroscopy results proved that petroleum coke can become an important low-cost precursor to scalable graphene production (Fig. 5c).

According to the same ecologically feasible precepts, Luong *et al.* (2020) developed a rapid synthesis technique similar to electrospun graphene using different carbon sources, among them, petroleum coke.^70^ Even though this mechanism promotes a turbostratic graphene arrangement, the study showed that calcined petroleum coke (when exposed to a high-voltage electrical discharge) works well as a source of non-graphitized carbon for the fast and high-yield production of ∼17 nm sized graphene and is potentially applicable in composites for building materials. In addition, Saikia *et al.* (2020) reported on the attainment of graphene nanosheets from petroleum coke using a hydrothermal method. From photocatalytic assays, nanostructures containing a high carbon content are shown to be an attractive alternative in the depollution of harmful 2-nitrophenols in wastewater systems.^71^

The use of petroleum coke is not only limited to the pure graphene synthesis. He *et al.* (2019) fabricated nitrogen- and oxygen-doped porous graphene from petroleum coke by a combination of urea-assisted high speed ball milling and annealing.^44^ The high specific surface area and heteroatom-rich structure promoted beneficial synergistic effects for catalytic performance of the aromatic compound desulfurization. Analogous to the material produced by He *et al.* (2019), Liu *et al.* (2020) used green petroleum coke to synthesize graphene-like plates doped with nitrogen and melanin. Their investigation demonstrated that petroleum green coke-based graphene was an excellent adsorbent for the removal of bisphenol-A from wastewater.^72^ In another study, Mandal *et al.* (2021) also used the liquid-phase exfoliation method with the ultrasonication process of petroleum coke to obtain sulfur and phosphorus-doped graphene sheets. They proved that graphene obtained from petroleum coke showed excellent electrochemical performance and energy storage capacity, properties that are applicable to supercapacitors.^73^

It is known that CNMs have a high surface area, excellent electrical conductivity, and chemical stability that enable applications in various fields, including energy conversion, storage devices and sensing. On the other hand, depending on the synthesis approach and carbon precursor to be used, different electrochemical responses may result. For example, Saha *et al.* (2021) produced a graphene derived from needle coke with a high electrical conductivity (∼56.9 S m^−1^) using the ECE method. However, even after a thermal annealing process, this same nanomaterial achieved an electrical conductivity of 250 S m^−1^, which is different and lower than the 9735 S m^−1^ value of graphene derived from graphite using the same synthesis approach.^10^ Another electrochemical response for potential energy storage applications is the specific capacitance, which is one of the important parameters for supercapacitors.^74^ Singaramohan *et al.* (2023) demonstrated that graphene derived from petcoke through ECE synthesis can be used as a supercapacitive material, showing a maximum specific capacitance of 40 F g^−1^ at a scan rate of 25 mV s^−1^ for pet graphene used in the H_2_SO_4_ electrolyte system.^75^ Unlike the CNM obtained by Mandal *et al.* (2021), using the same scan rate and H_2_SO_4_ electrolyte system, the graphene obtained by the liquid-phase exfoliation method of petroleum coke resulted in a specific capacitance of ∼48 F g^−1^.^73^

Based on this scenario, it can be seen that the electrochemical performance of CNM varies not only with the processing conditions, but also with the type of carbon raw material used. Furthermore, the morphological properties of carbon materials (such as crystallinity, the presence of heteroatoms, pore sizes, thicknesses and densities) all have an effect on electrochemical performance.^76–78^ It is based on these aspects that petroleum coke differs from other carbonaceous precursors, such as graphite, which has a structure made up of stacks of the highly oriented hexagonal layers of sp^2^ carbon. This non-calcined byproduct (green petroleum coke) has an ill-defined molecular structure, as a large part of this structure is composed of a complex arrangement of hydrocarbons, containing metallic impurities and high porosity.^79^ These characteristics result in less efficient electrochemical properties. For this reason, the high temperature treatment process permits the removal of impurities, as well as the removal of sulphur and covalently bonded oxygenated functional groups, restructuring the molecules in such a way as to increase the graphitization degree of synthesized nanomaterials,^10,80,81^ contributing positively to its electrochemical performance.

Assuming that petroleum coke is an alternative resource in obtaining graphene, a limited number of works involving these two materials were observed. This aggravating factor may be correlated to the petrochemical byproduct structure, since petroleum coke is made of stacked, linear, curved, and heteroatom-filled PAHs and defects.^79^ Acquiring an ordered lattice of atoms as a base structure of graphene from petcoke by means of current synthesis techniques becomes a major challenge. Thus, one way to solve this difficulty is to fuse and isolate the PAHs to form graphene nanosheets, and to cut them in the most defective regions to produce carbon quantum dots.^82^

### Carbon quantum dots (CQDs)

Among numerous studies of nanomaterials, carbon quantum dots (CQDs) belong to a class of 0D nanomaterials with particle sizes smaller than 10 nm, whose main characteristic is linked to its fluorescence.^83^ Due to their relative thermal stabilization, non-toxic nature, easy synthesis route and low cost,^84^ CQDs have been gaining prominence as the basis for light emitting devices,^85^ supercapacitor electrodes,^86^ in the manufacturing of military textiles,^87^ and in catalytic processes^88^ and sensors^89^ to name just a few.^90–92^

The harnessing of waste to design them as nanomaterials also applies to CQDs synthesis, especially for petroleum coke, which has been studied as an important precursor for this type of nanomaterial 0D. Wu *et al.* (2021) were able to synthesize hydrophilic CQDs with an average particle size of 2.54 nm through an electrochemical approach using petroleum coke as a carbon resource.^93^ They observed that treatment with ozone allowed the CQDs to achieve improved oil recovery in tight oil reservoirs without the use of surfactants.

Another study demonstrated the ability of petcoke to promote biomarker 0D nanostructures. Ma *et al.* (2020) reported the decomposition of petroleum coke by supercritical carbon dioxide, obtaining different feedstock sizes and generating different colors of CQDs by hydrothermal treatment.^94^ In this study, confocal microscopy results demonstrated cell viability and the presence of red fluorescence in macrophages exposed to treated petroleum coke CQD, indicating that the nanomaterial could be safely applied in environmental, energy and biomedical fields. In addition to being a great biomarker, CQDs are susceptible to sensing applications. Wang *et al.* (2015) reported the yellow fluorescence of petroleum coke-derived CQDs synthesis through ultrasound-assisted chemical oxidation, where they were used to detect Cu^2+^ ions in water.^95^ They concluded that Cu^2+^ detection is related to the fluorescence quenching of CQDs synthesized by photoinduced electron transfer mechanism, which allows the yellow fluorescent CQD probes to have a linear detection range of 0.25 to 10 μM, a detection limit of 0.0295 μM, and a response time of 3 s.

**Fig. 6:**
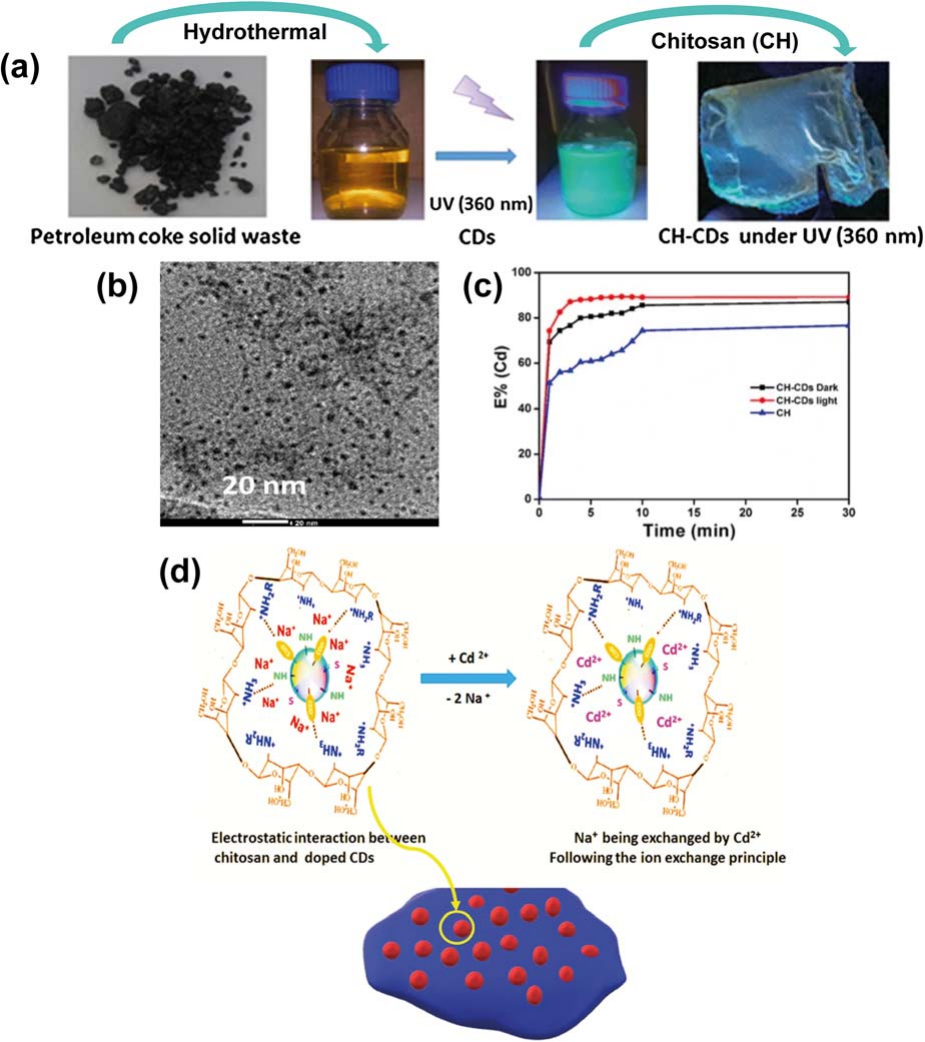
(a) Synthesis process of CQDs and CQDs-CH from petroleum coke residues, (b) TEM image of CQDs, (c) extraction efficiency at different times for Cd^2+^ using CH and CH-CDs in the dark and under UV light (360 nm) at pH = 8, and (d) mechanism of cation exchange in the presence of Cd^2+^ of the CH-CDs membrane. Reproduced (adapted) with permission from ref. 97. Copyright© 2020, Environmental Sciences Europe.

Liu *et al.* (2017) produced a N-doped graphene/CQD nanocomposite as an efficient catalyst in the oxygen reduction reaction (ORR) present in fuel cells. The CQDs with an average diameter of 2–4 nm used in the study were synthesized by acid oxidation of petroleum coke as the main precursor.^96^ Similarly, Jlassi *et al.* (2020) also synthesized CQDs *via* acid oxidation of petroleum coke (Fig. 6a).^97^ With an average size of 5 nm (Fig. 6b), the N and S-doped CQDs subsequently were dispersed in a chitosan polymeric matrix, forming a hybrid film for heavy metal removal. The presented work demonstrates that besides being a water-soluble, biocompatible and photoluminescent material over a wide pH range, it is also an excellent metal ion adsorbent, especially for the removal of Cd^2+^ ions (Fig. 6c and d), paving the way for adsorbent films of CQDs in water treatment using low-cost precursors from petroleum coke.

The co-doping of petroleum coke-derived CQDs is not limited to nitrogen and sulfur. Liu *et al.* (2021) synthesized boron and nitrogen co-doped CQDs by an electrochemical etching method using calcined petroleum coke electrodes.^98^ From the electrolytic solution of ammonia and sodium tetraborate, the boron and nitrogen species were incorporated into the 5.4 nm diameter CQD structure, exhibiting excellent ORR performance and better electrocatalytic stability than commercial Pt/C-type catalysts.

**Fig. 7:**
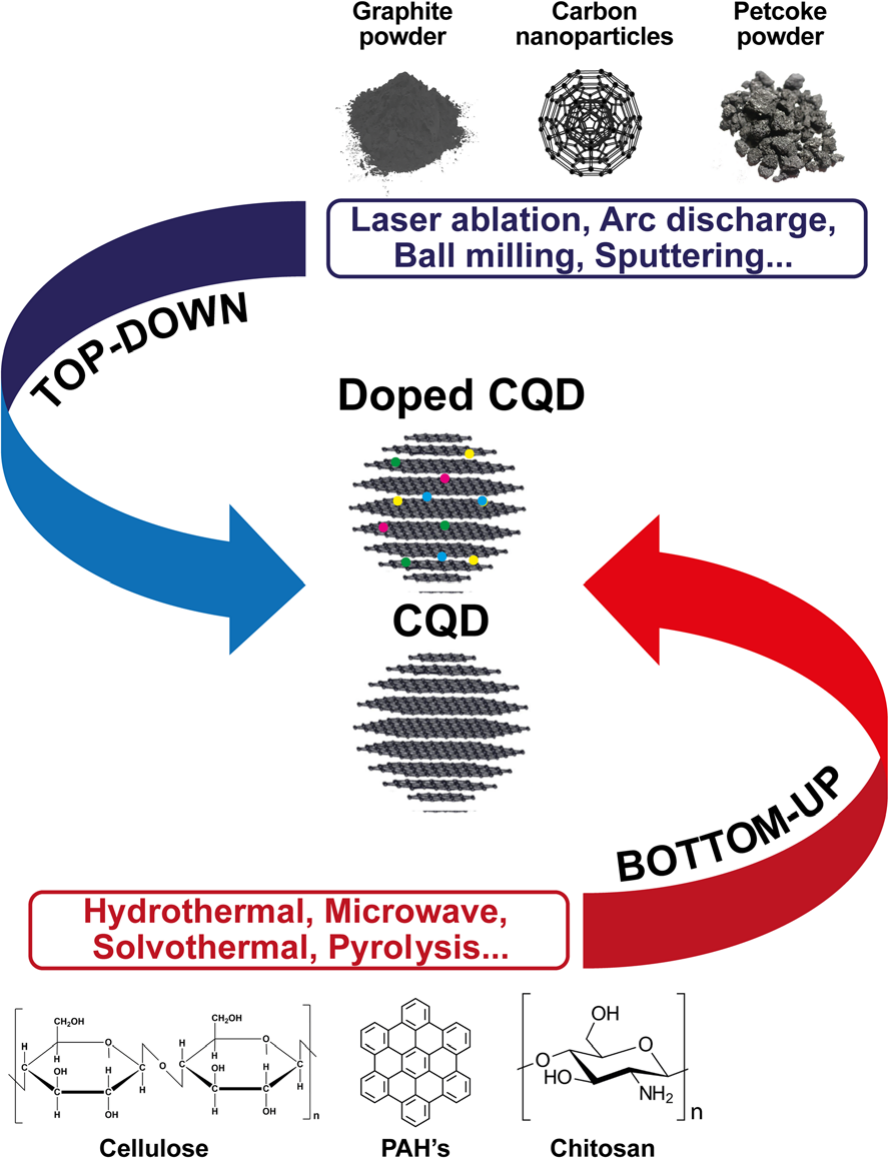
Synthesis methods of doped and un-doped CQD through top-down and bottom-up approaches.

Although many studies have been conducted into the various applications of heteroatoms-doped CQD in the carbon structure, including N, S, B, *etc.*, few studies have been published on doping with these and other chemical elements in petroleum coke-derived CQDs, especially when it comes to heteroatoms such as fluorine and phosphorus. This absence of further studies of heteroatoms-doped CQDs obtained from petroleum coke may be related to the synthesis approach used to generate CQDs, since obtaining heteroatoms-doped CQDs can be done through top-down and bottom-up approaches (Fig. 7). In the first route, doping already occurs in the carbonaceous material, or the doping occurs during the process of breaking/cleaving larger carbon structures (such as activated carbon, graphite powder, carbon nanoparticles, toluene and petroleum coke powder). Conversely, in the second route, the doped CQD is synthesized from the carbonization of small organic polycyclic aromatic hydrocarbon (PAH) molecules,^99^ chitosan, cellulose, biomass waste, *etc.*

However, the top-down approach requires equipment and more expensive materials, long reaction times, severe reaction conditions, as well as being a route that does not allow for precise control of the shape and size of the doped CQDs.^99,100^ These and other factors, such as the low yield methods and start from high-cost powdered materials, such as graphene^101^ and GO,^102^ suggest the bottom-up route as a more favorable approach for the synthesis of doped QDs because it allows for carbon conversion from smaller molecules through external energy application, such as hydrothermal method, solvothermal, microwave synthesis, electrochemical, *etc.*, and allows for safe synthesis conditions, as well as the effective control of the morphology and uniform size distribution of the nanomaterial.^103^ We can therefore infer that the small amount of published work on doping heteroatoms in CQDs derived from petroleum coke is due to the advantages provided by the bottom-up methods, and that it is more convenient to use heteroatoms in CQDs using this approach.^104^

Although graphite is a traditional precursor for obtaining different CNMs, structures similar to graphene and CQD that can be obtained from other carbon-rich sources, including rice husks,^105^ lignocellulosic waste,^106,107^ corn starch,^108^*etc.*, are promising for electrochemical applications. This is due to the intrinsic porosity of the raw material, which facilitates the penetration of electrolytic substances, reducing the diffusion distance of ions, as well as the presence of nitrogen generally contained in the biomass and other heteroatoms that contribute to the reactions through larger quantities of active sites.^109^ However, in contrast to petroleum coke-derived CNMs whose complex structures of aromatic hydrocarbons are dependent on the source and type of coking, obtaining CNMs derived from plant materials is dependent on their lignocellulosic structure. The plant carbonization materials contain fewer crystalline regions in their structure, resulting in spherical CNMs with low porosity, such as CQDs. On the other hand, plant materials with more crystalline regions, under the same processing conditions, result in CNMs with high porosity because they are able to preserve their structural characteristics after carbonization.^109^

Therefore, most CQD properties depend strongly on the original precursor.^110^ In addition to the origin source, we can infer that the CQDs properties, especially fluorescence, are influenced by the quantum confinement linked to the particle size,^111^ and by various functional groups contained in the structure on the carbon core surface (surface defects) after oxidation during and after synthesis, such as carbonyl, hydroxyl, carbonyl, sulfoxides, *etc.*^112^ Functional groups have different energy levels that can be related to their ability to supply electrons. Thus, when light of a specific length is directed on the CQD, the photons whose energy satisfies the optical bandgap promote the formation and capture of excitons that contributes to both photoluminescence phenomena (when electrons return to the fundamental state to emit visible light of different wavelengths) and photooxidative performance, where it allows the production of reactive oxidative species (ROS) (*e.g.*, O_2_, H_2_O_2_, OH*) in an aqueous medium that degrade organic compounds.^113,114^ Therefore, as the level of oxygenation increases during synthesis, the greater presence of surface defects retains a greater amount of excitons, which will largely determine the CQDs properties.

### Graphene quantum dots (GQDs)

Although graphene quantum dots (GQDs) and carbon quantum dots (CQDs) have a large surface area, low toxicity, excellent stability and are chemically composed mainly of carbon,^115^ GQDs and CQDs differ in their structural properties. This is because GQDS have a crystalline lattice formed by sp^2^ bonds containing point-centered graphene sheets with less than 10 nm particle size and are only 10 layers thick, while CQDs are constituted of an amorphous structure with sp^3^ hybridizations of almost spherical shape.^116^ Thus, GQDS exhibit exceptional electronic and optical properties as a consequence of quantum confinement,^117^ making this nanomaterial excellent in the UV-Vis absorption spectrum, exhibiting stable fluorescence, photoluminescence, among other enhanced properties^118^ that serve as a basis for supercapacitors,^119^ batteries,^120^ catalysts,^121^ biomarkers,^122^ photocatalytic water splitting and CO_2_ reduction,^123^ among others.^124–126^

Its use in several applications led researchers to investigate different carbon sources as an economical and sustainable alternative to synthesize GQDs, since they reduce waste generation and dependence on non-renewable carbon sources, such as graphite. Thus, petroleum coke is an alternative resource for GQDs production. For example, Thomas and Balachandran (2023) reported on biomarkers based on petroleum coke-derived GQDs using Hummers' improved method, using ethylenediamine and phosphoric acid as nitrogen and phosphorus doping agents, respectively.^127^ It was found that N-doped GQDs had better photophysical characteristics than F-doped GQDs or both heteroatoms, showing an increase in quantum yield (16%), greater photostability (92%), a fluorescence lifetime of 8.51 ns, as well as a cell viability of ∼85% even after 24 h of incubation, demonstrating that petroleum coke-derived N-GQD can be a cancer cell biomarker for *in vitro* imaging applications, which is essential in cancer diagnosis and therapy.

Yew *et al.* (2017) used calcined petroleum coke as a carbon source for the synthesis and attainment of GQDs by the oxi-reduction method with acidic agents. Due to their excellent photoluminescent properties, the petcoke-derived GQDs exhibited excellent performance in the detection of DNA at low concentrations (0.004–4 nM), showing its potential as a promising material for the development of affordable and efficient nanobiosensors in the detection of highly sensitive nucleic acid.^128^

Ye *et al.* (2013) described the direct synthesis of GQDs from three pregraphitic sources, one of them being petroleum coke, which was sonified in a sulfonitrile solution and thermally treated, resulting in GQDs of 6 nm and yield of 10–20 (wt%).^129^

### Carbon nano-onions (CNOs)

Another type of CNMs classified as a zero-dimensional nanostructure is CNOs or bucky onions, with particle sizes ranging from 5–100 nm. This nanomaterial similar to an onion cut in half is a part of the family of fullerenes that are formed, for the most part, during the thermal annealing of nanodiamonds at high temperatures under an inert atmosphere in a high vacuum.^130^ Depending on the preparation method, the carbon atoms rearrange themselves into quasi-spherical and polyhedral graphitic layers, forming a fullerene core encircled by concentric graphene layers^130^ with *d*-spacing approximately equal to the distance between two graphitic planes (0.334 nm).^131^

Although CNOs can present different shapes (spherical^132^ and polygonal^133^), diameters (small^134,135^ and large^132^) and cores (dense^135^ or hollow^134^), depending on the preparation method used, CNOs have similar structures, composed of carbon atoms arranged in hexagonal and pentagonal rings located at the vertices of these structures, and forming two single bonds and one double bond around carbon atoms. These bonds result in delocalised p electrons in the structure,^130^ which confer exemplary electrochemical properties as high-performance materials for energy storage.^136^ Moreover, CNOs can be produced from various hydrocarbon sources, which include plant extracts (tomato),^137^ castor oil,^138^ butter,^139^ organic gases,^140^ agricultural^141^ and plastic^142^ wastes, among others. However, petroleum coke is not left out of this list.

Recently, Lei *et al.* (2021) proved spherical CNOs formation with a petroleum coke core decorated with pitch, both used as carbon sources, respectively.^135^ The mixture pyrolysis of the two raw materials under atmospheric nitrogen gas conditions produced CNOs of sizes between 5 and 30 nm in diameter that provided a high surface area and abundant volume of micropores present in the structure. Pyrolysis is a method that consists of thermal degradation of the organic material in the partial or total absence of an oxidising agent, or even in an environment with an oxygen concentration capable of preventing the intensive gasification of the organic material. Moreover, electrochemical analyses of CNOs investigated in an electrode system showed a high specific capacitance at 312 F g^−1^ at a current density of 1 A g^−1^, and a low ohmic resistance of 0.42 Ω and good energy density of 7.47 W h kg^−1^ at the power density of 221 W kg^−1^.^135^ Such results suggest that petrochemical waste can be converted into CNMs with high capacitance and excellent electron and ion transfer rate, fundamental requirements for practical application in high-performance supercapacitors.

### Graphene oxide (GO)

Graphene oxide is one of the carbon allotropes that consists of a flat layer of carbon atoms organized in a hexagonal network filled with functional groups containing oxygen in its basal plane and at the edges in epoxy, hydroxyl and carboxyl forms, which result in a mixture of sp^2^ and sp^3^ hybridized carbon atoms.^143^ This chemical configuration allows covalent or non-covalent interactions with various other molecules,^144^ which makes GO a promising resource for material production in catalytic systems for water purifying,^145^ as approached in a study by Almarzooqi *et al.* (2021) who prepared and tested GO-coated polyethylene terephthalate membranes for the desalination and separation of produced water,^146^ as well as biosensing^147^ and drug delivery for the treatment of genetic diseases.^148^ In addition, the high electrical thermal conductivity, high surface area and high mechanical strength of GO make possible various applications, including optoelectronic devices,^149^ supercapacitors,^150^ and base material for reinforcement in composites.^151^

**Fig. 8:**
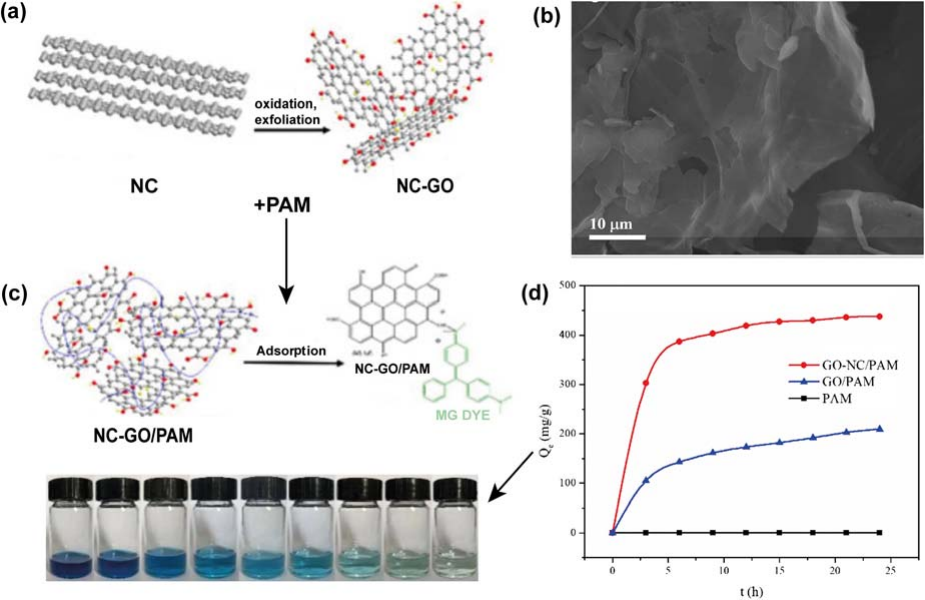
(a) Synthesis process of GO derived from petroleum coke (GO–NC), (b) SEM image of GO–NC, (c) process of obtaining the PAM/GO–NC hydrogel and adsorption of the MG dye, and (d) adsorption capacity of GO–NC/PAM, GO/PAM and PAM. Reproduced (adapted) with permission from ref. 152. Copyright© 2018, Taylor & Francis.

The most common way to produce GO is realized through graphite oxidation, followed by exfoliation of this oxide using acid agents. However, this mineral is not the only precursor for obtaining GO nanosheets. Thus, petroleum coke is also considered as an alternative precursor for the manufacturing of this 2D nanomaterial. Xing *et al.* (2019) carried out the synthesis of GO nanosheets through the oxi-reduction process following a modified Hummers' method using needle coke (GO–NC) (Fig. 8a).^152^ In addition to the morphology of ultrasonically exfoliated GO–NC (Fig. 8b) being analogous to graphite precursor-based GO nanosheets, the researchers produced polyacrylamide (PAM)/GO–NC hydrogels (Fig. 8c), which resulted in a nanocomposite with high adsorption capacity for the malachite green dye (Fig. 8d).

In another study, Sierra *et al.* (2016) demonstrated that pre-graphitic materials from different origins can be used as graphene oxide precursors.^153^ In this work, ultrasonically exfoliated GO sheets generated from oxidized petroleum coke exhibited similar characteristics to those reported for other GOs obtained from graphite in terms of the defects, type, and content of oxygen functional groups. During the same period, Sierra *et al.* reported on the GO formation prepared from petcoke by Hummers' method, followed by the sonication technique.^154^ From the Raman spectroscopy results, the GO derived from petcoke oxidation provided less decoration of oxygenated groups present in the basal plane.

### Reduced graphene oxide (RGO)

Reduced graphene oxide (RGO) is a 2D nanomaterial prepared by chemically oxidizing natural graphite to obtain graphene oxide, and then reducing it to the point of producing a material with a low content of oxygen functional groups present in the basal plane of graphite. As such, RGO has attracted research attention due to its intrinsic properties that are similar to those of graphene, such as biocompatibility,^155^ high mechanical resistance,^156^ high surface-to-volume ratio,^157^ and high thermal and electrical conductivities.^158^ Furthermore, the oxidation and hydrothermal methods of GO to generate RGO are less expensive than the current methods of graphene synthesis, which are quite expensive and of low yield, such as mechanical exfoliation and CVD. However, the electronic and mechanical properties of RGO present substantially reduced values when compared to graphene.^159^

Generally, graphite is the most used precursor for the RGO preparation process because it has crystalline phases that provide the basal structural unit of graphene.^67^ However, the mechanisms are not limited only to this mineral. Considering that the high carbon content in raw material is an important parameter to use it in CNMs preparation, it is not clear whether petcoke is suitable as a precursor of RGO, because its production process from this by-product is more challenging in view of the PAH structural nature encrusted oxygen functional groups present in petroleum coke,^79^ which may affect the quality and efficiency of the post-reduction final product, as RGO has a larger amount of defects than graphene.^160^

Therefore, it is observed that few works have been reported on using petroleum coke as a carbon source for RGO synthesis over the years. However, considering the feasibility and economy, coke can become a prime candidate in the nanomaterial production, bringing added value. Kumar *et al.* (2023)^161^ used GO derived from thermally pre-treated PC to produce RGO by the Hummers' method, followed by a hydrothermal reduction. From these procedures, they were able to obtain a carbon-based nanostructure with remarkable electrocatalytic properties in terms of stability, efficiency, and large-scale production of green H_2_ as a sustainable fuel source. Furthermore, XRD analysis confirmed the presence of graphitic carbon in petroleum coke after heat treatment above 2000 °C, suggesting that treatment converts the amorphous carbon of pure petroleum to a more crystalline densified structure due to the removal of pores and void reduction that results in a smaller band gap, which is ideal for improving the electrocatalytic performance for hydrogen and oxygen evolution reactions (HER and OER). This makes PC a candidate material for producing more valuable products and for application in energy devices.

Similar to how Xing *et al.* (2019) synthesized GO from needle coke, the researchers used the same precursor to produce RGO nanosheets by a thermal reduction method of GO.^152^ As reported, thermal reduction of GO provided RGO nanosheets with smaller than ten graphene layers with a basal graphite crystal structure. It is important to emphasize that ordered graphitic structures can achieve fast charge transfer characteristics conditioned by their sp^2^ hybridized structure, contributing positively to the enhancement of their supercapacitive properties under high current density conditions.^162^

### Carbon nanotubes (CNTs)

CNTs is one of the most remarkable CNMs of the 1D nanostructure family, as they exhibit excellent mechanical properties, high thermal and electrical conductivities,^163^ as well as electrochemical properties,^164^ such as enhanced voltammetric currents,^165^ high heterogeneous electron transfer, electrocatalytic effect in H_2_0_2_ production^166^ and fouling-resistant electrodes.^167^ As a result, CNTs have attained the focus of innumerable works for a wide range applications that from electronic devices,^168^ biosensors,^169^ biomedicine,^170^ photovoltaic cells,^171^ to drug delivery^172^ and removal organic pollutants/heavy metals.^173^

One of the factors that justify the excellent properties of CNTs is their structure which consists of sp^2^ hybridized carbon atoms, arranged in a benzene rings progression, forming a rolled graphene sheet in a cylindrical shape with an external diameter of <100 nm, which can have an open or closed end.^173^ Thus, CNTs can be classified according to the number of layers present in their structure, so that single-walled carbon nanotubes (SWCNTs) are characterized by a single rolled graphene sheet, while double-walled carbon nanotubes (DWCNTs) or multiple-walled carbon nanotubes (MWCNTs) consist of two or more graphene sheets involving a hollow core analogous to SWCNTs.

In all methods for CNTs synthesis, energy and carbon sources are used. For example, arc discharge, laser ablation and chemical vapour deposition use different carbon sources that include methane, acetylene, benzene, xylene, and toluene,^174^ presuming that the choice of carbon source can affect the properties of CNTs, such as the diameter, length and purity degree. However, an important factor to consider is the cost of the raw material used in CNTs synthesis. Although the carbon sources mentioned are common, they are also relatively expensive. This may limit the CNTs application in certain areas due to the high cost of production. Therefore, it is necessary to consider the development of this nanomaterial from cheaper raw materials in industrial scale production to increase its competitiveness before other means.

In this context, researchers have explored other organic carbon sources for CNTs synthesis, in addition to those mentioned above. Some of these sources include essential and vegetable oils,^175,176^ chicken feathers,^177^ plastics^178^ and agricultural wastes,^179^ biodiesel,^180^ as well as petroleum hydrocarbon. As an example, Xu *et al.* (2014) realized a study in which they synthesized SWCNTs and DWCNTs by arc discharge in a controlled manner using petroleum coke.^181^ The method was executed under atmospheric He and Ar gas conditions, resulting in a content of SWCNTs and DWCNTs (Fig. 8a) with a purity above 95% and average outer diameter distribution concentrated between 1 and 1.6 nm (Fig. 8b) and 3–4.4 nm (Fig. 8c), respectively. Moreover, they fabricated solar cells based on films of these CNTs under single-crystal silicon substrates, and observed that SWCNTs-based solar cells (Fig. 8d) exhibited higher energy conversion efficiency compared to DWCNTs-based solar cells (Fig. 8e) under LED illumination in the range between 400 and 940 nm. However, it was noted that the performance can be improved from the combination of both, demonstrating that petroleum coke is a viable feedstock that can be converted into different types of high quality and value-added CNTs.

### Nanoporous carbon

The synthesis of petroleum coke-derived nanoporous carbon involves a heating process of the precursor at high temperatures under a controlled atmosphere, followed by activation steps to create the desired nanoporous structure. Generally, the coke oxidation can be carried out in an acidic solution, in a mixture of HNO_3_ and H_2_SO_4_. In addition, the activation of these nanopores can be carried out using physical methods, such as heat treatment in the presence of inert gases, or chemical methods using activating agents, such as KOH or H_3_PO_4_. These activation procedures promote the removal of volatile components that are commonly in petroleum coke, resulting in a porous carbonaceous structure with a high proportion of nanopores.^182^ Greater porosity favors the diffusion of reagents and/or adsorbents, both internally and on the materials surface, as well as provides a high surface area that increases their capacity for adsorption and interaction with molecules and ions. During synthesis, it is possible to adjust the size, shape and distribution of nanopores carbon.^182^ Various structures and morphologies of nanoporous carbons can be produced using a variety of carbon precursors. Characteristics, such as the composition and structure of the precursors, have a crucial role in controlling the resulting nanoporous carbons properties. Some research has shown that various source materials, such as coal, biomass (including agricultural waste) and petrochemical compounds, have been used in different types of synthesis methods of the nanoporous carbons, each with their own distinct surface properties.^183^ Nanoporous carbon has intrinsic chemical/thermal stability, and is resistant to adverse chemical environments and high temperatures, giving this material durability. In certain applications, carbon nanopores can exhibit satisfactory electrical conductivity, which makes them suitable for use in electrochemical devices such as supercapacitors and fuel cells.^184^ Thus, petroleum coke-derived nanoporous carbons have been widely studied in supercapacitor development with a high capacitive retention rate, high energy, power density, excellent cycling stability and coulombic efficiency,^26^ including lithium-ion capacitors,^3^ Zn-ion hybrids (ZICs).^185^ Other applications have been successfully implemented in the capture of CO_2_ from flue gases.^186^

## Market

**Fig. 9:**
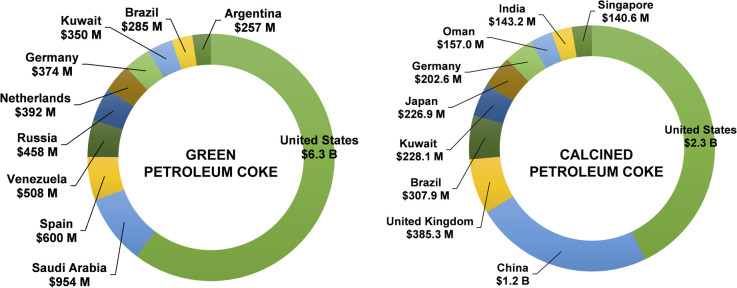
The top exporters of green petroleum coke and calcined petroleum coke in 2022.^187^

Approximately 75% of the worldwide production uses petcoke as a fuel source, while the rest is directed toward the manufacturing of products such as steel and electrodes.^13^ In this context, petroleum coke, including green and calcined, is a worldwide traded commodity with financial returns generating $18.72 B in 2022.^187^ The United States, Canada and Singapore and China are the largest exporters of this sub-product, as shown in Fig. 9.

Furthermore, needle coke used in electrodes in steel production can be purchased for ∼$1500–3000 per ton. Bulk graphene powder can be purchased on a laboratory scale for ∼$8 per gram, which is orders of magnitude more valuable than coke. Other data show that global needle coke production was 1.1 million tons per year in 2020, and is expected to increase to 1.5 million tons per year until 2026.^10^ However, this estimate refers to demand for the steel and lithium-ion battery production industries,^10^ which can be increased if it is destined to graphene and other CNMs production, since the financial capital from the commodity commercialization tends to increase in value in an industry 4.0 scenario, where the global nanotechnology market is projected to reach more than US $91.8 billion by 2028.^188^

Understanding the dynamic nature of the CNMs pricing is essential to appreciate the interaction complex with their sources, especially when it is related to petroleum coke, as the byproduct prices are influenced by a variety of factors, including supply and demand dynamics, money supply, production, competition, interest rates, political factors, among others.^189–191^ Therefore, considering that a greater supply of other carbon precursors is present on the market, in addition to the traditional raw material used for the production of graphite-based nanomaterials, the prices of CNMs can be affected based on the premise of the law of supply and demand. For example, the increase in demand for lithium-ion batteries caused the Chinese industry to increase its graphitization capacity, exceeding the 2.3 million tons of graphite produced in the last period of 2022 compared to 1 million tons in March of the same year. This situation was also attributed to the significant drop of more than 40% in the price of needle-type petroleum coke, an important source of raw material for the production of synthetic graphite, when compared to 2021.^192^

It is interesting to realize that since graphene was first discovered in 2004 by A. K. Geim and K. S. Novoselov,^193^ a vast number of scientific papers and patents have been produced, reaching over 50 000 patents filed through May 2019 referring to energy applications,^188^ for example. However, natural graphite is a finite source with an estimated 800 million tons, meaning that a small portion of this amount is basal graphitic carbon for nanostructures production.^194^ Therefore, it is very important to expand the set of precursors of CNMs to include other industrial byproducts, such as petcoke and its subtypes, thus making it more commercially attractive.

GO is still a product with a low market share, but the projection for the coming years is that its demand will only go up, thus making it a more attractive product for investors. According to the report “The Global Graphene Market,” released in 2019 by the consulting firm Research and Markets, it reports there are 200 companies in the world that produce materials that use graphene or are developing products that incorporate graphene, mostly in North America, Europe, China, and Australia. This market is estimated to grow 30% per year and reach $250 million by 2025.^195^

Another aggravating factor that supports the potential market for the use of petcoke in the production line of CNMs is also linked to the environmental impacts. In addition to the problems previously mentioned in this study, the generation of fugitive dust from petroleum coke piles is an eminent concern as the global stockpile of this petrochemical byproduct has been increasing considerably at a rate of ∼6 million tons per year,^196^ which can lead to the accidental inhalation of organisms in localities near residential areas. About 100 tons of petroleum coke fugitive dust is released into the atmosphere per year in the United States.^197^ These and other concerns reported earlier press the need for the management of petcoke for sustainable and cost-effective end uses, as a precursor to CNM production.

## Conclusions

In general, petroleum coke is commercialized in green or calcined form, depending on the refining quality specifications and the source of crude oil, with different types, such as needle coke, shot coke and sponge coke. There are different fields of application for petroleum coke, mainly in the area of energy generation and in the aluminum smelting process as a carbon anode. On the other hand, its physicochemical properties, high carbon content, availability and low cost allow also use it as a precursor to obtain different CNMs, such as graphene, its derivatives (GO and RGO), and other 0D, 1D and 2D nanomaterials, in accordance with a defined synthesis method, where it was observed that the bottom-up approach is most advantageous for doped and undoped CQDs derived from petroleum coke.

However, there is a limited amount of published work compared to other carbon-based precursors, such as graphite, biomass and other carbonaceous residues. These studies are mainly aimed at influencing the synthesis parameters, the type of coke used, the phenomena and reaction mechanisms in the formation of different nanostructures, and how this entire set will interfere with the electrochemical performance and other properties related to CNMs. Therefore, there is a range of petroleum coke possibilities to be explored.

Furthermore, the production of petroleum coke-derived CNMs could bring both economic and socio-environmental benefits, considering that petrochemical industries are looking for cheaper resources, which promote value-added materials and satisfy the sustainable policies of the Sustainable Development Goals. Therefore, the future of petroleum coke as a precursor for the preparation of CNMs is very promising due to the benefits that its use can bring, not only because of its intrinsic microstructural characteristics that promote several applications for carbon nanostructures, including energy conversion, storage of energy, sensing, advanced oxidative processes, luminescence, catalysis, *etc.*, as well as because it is an abundant, low-cost source. Furthermore, in the last five years, there has been a significant effort in research focused on the exploration of CNMs obtained from petcoke, indicating a growing interest and potential for advances on the horizon in this field.

## Author contributions

Rivaldo Leonn Bezerra Cabral: conceptualization, data curation, methodology, formal analysis, investigation, writing—original draft preparation and writing—review and editing. Edney Rafael Viana Pinheiro Galvão: formal analysis, funding acquisition, project administration, validation, visualization, writing—review and editing. Pierre Basílio Almeida Fechine: formal analysis, validation, and writing—review and editing. Felipe Mendonça Fontes Galvão: visualization and writing—review and editing. José Heriberto Oliveira do Nascimento: formal analysis, validation, visualization, supervision and writing—review and editing.

## Conflicts of interest

There are no conflicts to declare.

## References

[cit1] Aitani A. M. , Encyclopedia of Energy, 2004, 4, 715–729

[cit2] Lim C., Lee J. (2020). Energy Policy.

[cit3] Veluri P. S., Katchala N., Anandan S., Pramanik M., NarayanSrinivasan K., Ravi B., Rao T. N. (2021). Energy Fuels.

[cit4] Chen J., Lu X. (2007). Resour., Conserv. Recycl..

[cit5] Sato A. K. C., Paulino R. F. S., de Campos V. A. F., Tuna C. E., Silveira J. L. (2022). Fuel.

[cit6] Tyutrin A. A., Burdonov A. E., Bushuev K. S. (2022). Mater. Sci. Forum.

[cit7] Wang M., Wan Y., Guo Q., Bai Y., Yu G., Liu Y., Zhang H., Zhang S., Wei J. (2021). Fuel.

[cit8] Maxim L. D., Galvin J. B., Niebo R., Segrave A. M., Kampa O. A., Utell M. J. (2006). Inhalation Toxicol..

[cit9] Lee S. M., Lee S. H., Park S., Yoon S.-H., Jung D.-H. (2022). Desalination.

[cit10] Saha S., Lakhe P., Mason M. J., Coleman B. J., Arole K., Zhao X., Yakovlev S., Uppili S., Green M. J., Hule R. A. (2021). npj 2D Mater. Appl..

[cit11] Li W., Wang B., Nie J., Yang W., Xu L., Sun L. (2018). Energies.

[cit12] Liu X., Zhou Z., Hu Q., Dai Z., Wang F. (2011). Energy Fuels.

[cit13] Caruso J. A., Zhang K., Schroeck N. J., McCoy B., McElmurry S. P. (2015). Int. J. Environ. Res. Public Health.

[cit14] González A., Moreno N., Navia R. (2014). Chemosphere.

[cit15] U. Nations , Transforming Our World: The 2030 Agenda for Sustainable Development (A/RES/70/1), https://sdgs.un.org/2030agenda, accessed 17 May 2023

[cit16] Gottardo S., Mech A., Drbohlavová J., Małyska A., Bøwadt S., Riego Sintes J., Rauscher H. (2021). NanoImpact.

[cit17] Gray M. R. , Upgrading Oilsands Bitumen and Heavy Oil, University of Alberta, 2015

[cit18] Briens C., McMillan J. (2021). Energy Fuels.

[cit19] Parkash S. , Petroleum Fuels Manufacturing Handbook: Including Specialty Products and Sustainable Manufacturing Techniques, McGraw-Hill Education, 2010

[cit20] Administration U. S. E. I. , Petroleum and Liquids: Number and Capacity of Petroleum Refineries, https://www.eia.gov/dnav/pet/pet_pnp_cap1_dcu_nus_a.htm, accessed 14 October 2023, p. 2023

[cit21] Gray M. R. (2002). Can. J. Chem. Eng..

[cit22] Guerta A. C., Peres C. B., de Campos V., Yamaji F. M., Cardoso de Morais L. (2023). Solid Fuel Chem..

[cit23] Andrews A. and Lattanzio R. K., Petroleum Coke: Industry and Environmental Issues, Congressional Research Service, Washington, DC, 2013

[cit24] Edwards L. (2015). JOM.

[cit25] Yao J., Liu C., Zhu Y., Sun Y., Feng D., Li H., Yang Y., Ma T., Qiu J. (2024). Carbon.

[cit26] Cheng J., Lu Z., Zhao X., Chen X., Liu Y. (2021). J. Power Sources.

[cit27] Ibrahim H. A. (2017). Recent Advances in Petrochemical Science.

[cit28] Sato A. K. C., Paulino R. F. S., de Campos V. A. F., Tuna C. E., Silveira J. L. (2022). Fuel.

[cit29] Kääntee U., Zevenhoven R., Backman R., Hupa M. (2004). Fuel Process. Technol..

[cit30] Chen Z., Ma W., Li S., Wu J., Wei K., Yu Z., Ding W. (2018). J. Cleaner Prod..

[cit31] Shao W., Shi H., Jian X., Wu Z.-S., Hu F. (2022). Adv. Energy Sustainability Res..

[cit32] Dou X., Hasa I., Saurel D., Vaalma C., Wu L., Buchholz D., Bresser D., Komaba S., Passerini S. (2019). Mater. Today.

[cit33] Fan X., Kong X., Zhang P., Wang J. (2024). Energy Storage Mater..

[cit34] Yin J., Jin J., Chen C., Lei Y., Tian Z., Wang Y., Zhao Z., Emwas A., Zhu Y., Han Y., Schwingenschlögl U., Zhang W., Alshareef H. N. (2023). Angew. Chem., Int. Ed..

[cit35] Ebbesen T. W. , Carbon Nanotubes: Preparation and Properties, CRC Press, 1996

[cit36] Inagaki M. and Kang F., Materials Science and Engineering of Carbon: Fundamentals, Butterworth-Heinemann, 2014

[cit37] Liu M., Zhang X., Wu W., Liu T., Liu Y., Guo B., Zhang R. (2019). Chem. Eng. J..

[cit38] Nair R. R., Blake P., Grigorenko A. N., Novoselov K. S., Booth T. J., Stauber T., Peres N. M. R., Geim A. K. (2008). Science.

[cit39] Lee C., Wei X., Kysar J. W., Hone J. (2008). Science.

[cit40] Khanafer K., Vafai K. (2017). Int. J. Heat Mass Transfer.

[cit41] Bolotin K. I., Sikes K. J., Hone J., Stormer H. L., Kim P. (2008). Phys. Rev. Lett..

[cit42] Yu S., Wang X., Tan X., Wang X. (2015). Inorg. Chem. Front..

[cit43] Munz M., Giusca C. E., Myers-Ward R. L., Gaskill D. K., Kazakova O. (2015). ACS Nano.

[cit44] He J., Wu P., Lu L., Sun H., Jia Q., Hua M., He M., Xu C., Zhu W., Li H. (2019). Energy Fuels.

[cit45] Xu K., Li Y., Yang F., Yang W., Zhang L., Xu C., Kaneko T., Hatakeyama R. (2014). Carbon.

[cit46] Yan F., Sun Z., Zhang H., Sun X., Jiang Y., Bai Z. (2019). Microchim. Acta.

[cit47] Paternò G. M., Goudappagouda, Chen Q., Lanzani G., Scotognella F., Narita A. (2021). Adv. Opt. Mater..

[cit48] Hamidi Zirasefi M., Khorasheh F., Ivakpour J., Mohammadzadeh A. (2016). Energy Fuels.

[cit49] Zhai W., Zhou K. (2019). Adv. Funct. Mater..

[cit50] Bunch J. S., Verbridge S. S., Alden J. S., Zande A. M. (2008). Nano Lett..

[cit51] Hummers Jr W. S., Offeman R. E. (1958). J. Am. Chem. Soc..

[cit52] Zhang Y., Small J. P., V Pontius W., Kim P. (2005). Appl. Phys. Lett..

[cit53] Liu N., Luo F., Wu H., Liu Y., Zhang C., Chen J. (2008). Adv. Funct. Mater..

[cit54] Caicedo F. M. C., López E. V., Agarwal A., Drozd V., Durygin A., Hernandez A. F., Wang C. (2020). Diamond Relat. Mater..

[cit55] Behabtu N., Lomeda J. R., Green M. J., Higginbotham A. L., Sinitskii A., V Kosynkin D., Tsentalovich D., Parra-Vasquez A. N. G., Schmidt J., Kesselman E. (2010). Nat. Nanotechnol..

[cit56] Gonçalves G., Borme J., Bdkin I., González-Mayorga A., Irurueta G., Nogueira H. I. S., Serrano M. C., Alpuim P., Marques P. A. A. P. (2018). Carbon.

[cit57] Toh S. Y., Loh K. S., Kamarudin S. K., Daud W. R. W. (2014). Chem. Eng. J..

[cit58] Bourlinos A. B., Georgakilas V., Zboril R., Steriotis T. A., Stubos A. K. (2009). Small.

[cit59] Tang S., Jin S., Zhang R., Liu Y., Wang J., Hu Z., Lu W., Yang S., Qiao W., Ling L. (2019). Appl. Surf. Sci..

[cit60] Zhang W., Cui J., Tao C., Wu Y., Li Z., Ma L., Wen Y., Li G. (2009). Angew. Chem..

[cit61] Li X., Cai W., An J., Kim S., Nah J., Yang D., Piner R., Velamakanni A., Jung I., Tutuc E., Banerjee S. K., Colombo L., Ruoff R. S. (2009). Science.

[cit62] Abbasi E., Akbarzadeh A., Kouhi M., Milani M. (2016). Artif. Cells, Nanomed., Biotechnol..

[cit63] Li N., Wang Z., Zhao K., Shi Z., Gu Z., Xu S. (2010). Carbon.

[cit64] Choucair M., Thordarson P., Stride J. A. (2009). Nat. Nanotechnol..

[cit65] Pei S., Wei Q., Huang K., Cheng H.-M., Ren W. (2018). Nat. Commun..

[cit66] Zaaba N. I., Foo K. L., Hashim U., Tan S. J., Liu W.-W., Voon C. H. (2017). Procedia Eng..

[cit67] Bianco A., Cheng H.-M., Enoki T., Gogotsi Y., Hurt R. H., Koratkar N., Kyotani T., Monthioux M., Park C. R., Tascon J. M. D. (2013). Carbon.

[cit68] Hope J. T., Sun W., Kewalramani S., Saha S., Lakhe P., Shah S. A., Mason M. J., Green M. J., Hule R. A. (2020). ACS Appl. Nano Mater..

[cit69] Achee T. C., Sun W., Hope J. T., Quitzau S. G., Sweeney C. B., Shah S. A., Habib T., Green M. J. (2018). Sci. Rep..

[cit70] Luong D. X., V Bets K., Algozeeb W. A., Stanford M. G., Kittrell C., Chen W., V Salvatierra R., Ren M., McHugh E. A., Advincula P. A. (2020). Nature.

[cit71] Saikia M., Das T., Dihingia N., Fan X., Silva L. F. O., Saikia B. K. (2020). Diamond Relat. Mater..

[cit72] Liu Z., Wang Q., Zhang B., Wu T., Li Y. (2020). Molecules.

[cit73] Mandal D., Mahapatra P. L., Kumari R., Kumbhakar P., Biswas A., Lahiri B., Chandra A., Tiwary C. S. (2021). Emergent Mater..

[cit74] Iqbal M. F., Mahmood-Ul-Hassan, Ashiq M. N., Iqbal S., Bibi N., Parveen B. (2017). Electrochim. Acta.

[cit75] Singaramohan D. P., Ramanujam S., Veerasamy M., Thomas S. P., Natesan B. (2023). J. Electrochem. Sci. Eng..

[cit76] Thapaliya B. P., Luo H., Halstenberg P., Meyer III H. M., Dunlap J. R., Dai S. (2021). ACS Appl. Mater. Interfaces.

[cit77] Zhou X., Li H., Yang J. (2016). J. Energy Chem..

[cit78] An G.-H., Ahn H.-J. (2013). Carbon.

[cit79] Zhong Q., Mao Q., Zhang L., Xiang J., Xiao J., Mathews J. P. (2018). Carbon.

[cit80] Zhu F., Song W., Ge J., Wang Z., Huang Z., Li S., Wang M., Zuo H., Jiao S., Zhu H. (2023). Advanced Science.

[cit81] Franklin R. E. (1951). Proc. R. Soc. London, Ser. A.

[cit82] Hu H., Wu M. (2020). J. Mater. Chem. A.

[cit83] An Y., Liu C., Li Y., Chen M., Zheng Y., Tian H., Shi R., He X., Lin X. (2022). Int. J. Mol. Sci..

[cit84] Latief U., ul Islam S., Khan Z. M. S. H., Khan M. S. (2021). Spectrochim. Acta, Part A.

[cit85] Zhao Y., Yu L., Deng Y., Peng K., Yu Y., Zeng X. (2023). Ceram. Int..

[cit86] Inayat A., Albalawi K., Rehman A., Saad A. Y., Saleh E. A. M., Alamri M. A., El-Zahhar A. A., Haider A., Abbas S. M. (2023). Mater. Today Commun..

[cit87] Emam H. E., El-Shahat M., Hasanin M. S., Ahmed H. B. (2021). Cellulose.

[cit88] Guo Y., Zhang R., Zhang S., Hong H., Zhao Y., Huang Z., Han C., Li H., Zhi C. (2022). Energy Environ. Sci..

[cit89] Feng X., Zhang Y. (2019). RSC Adv..

[cit90] Jain S., Dilbaghi N., Singhal N. K., Kaushik A., Kim K.-H., Kumar S. (2023). Chem. Eng. J..

[cit91] Batabyal S. , Pradhan B., Mohanta K., Bhattacharjee R. R. and Banerjee A., 2023

[cit92] Mogharbel A. T., Abu-Melha S., Hameed A., Attar R. M. S., Alrefaei A. F., Almahri A., El-Metwaly N. (2023). Arabian J. Chem..

[cit93] Wu Y., Cao M., Zhao Q., Wu X., Guo F., Tang L., Tan X., Wu W., Shi Y., Dai C. (2021). Carbon.

[cit94] Ma J., Zhang L., Chen X., Su R., Shi Q., Zhao S., Xu Q., Xu C. (2021). Chin. Chem. Lett..

[cit95] Wang Y., Wu W., Wu M., Sun H., Xie H., Hu C., Wu X., Qiu J. (2015). New Carbon Mater..

[cit96] Liu H., Zhao Q., Liu J., Ma X., Rao Y., Shao X., Li Z., Wu W., Ning H., Wu M. (2017). Appl. Surf. Sci..

[cit97] Jlassi K., Eid K., Sliem M. H., Abdullah A. M., Chehimi M. M., Krupa I. (2020). Environ. Sci. Eur..

[cit98] Liu H., Liu Z., Zhang J., Zhi L., Wu M. (2021). New Carbon Mater..

[cit99] Kou X., Jiang S., Park S.-J., Meng L.-Y. (2020). Dalton Trans..

[cit100] Wang Y., Hu A. (2014). J. Mater. Chem. C.

[cit101] Feng Q., Xiao W., Liu Y., Zheng Y., Lin Y., Li J., Ye Q., Huang Z. (2018). Materials.

[cit102] Sun H., Ji H., Ju E., Guan Y., Ren J., Qu X. (2015). Chem.–Eur. J..

[cit103] Desmond L. J., Phan A. N., Gentile P. (2021). Environ. Sci.: Nano.

[cit104] Meng W., Bai X., Wang B., Liu Z., Lu S., Yang B. (2019). Energy Environ. Mater..

[cit105] Wang Z., Yu J., Zhang X., Li N., Liu B., Li Y., Wang Y., Wang W., Li Y., Zhang L. (2016). ACS Appl. Mater. Interfaces.

[cit106] Zhao H., Zhao T. S. (2013). J. Mater. Chem. A.

[cit107] Ekhlasi L., Younesi H., Rashidi A., Bahramifar N. (2018). Process Saf. Environ. Prot..

[cit108] Teymourinia H., Salavati-Niasari M., Amiri O., Farangi M. (2018). J. Mol. Liq..

[cit109] Matveeva V. G., Bronstein L. M. (2022). Prog. Mater. Sci..

[cit110] Tian Z., Zhang X., Li D., Zhou D., Jing P., Shen D., Qu S., Zboril R., Rogach A. L. (2017). Adv. Opt. Mater..

[cit111] Yuan T., Meng T., He P., Shi Y., Li Y., Li X., Fan L., Yang S. (2019). J. Mater. Chem. C.

[cit112] Shabbir H., Csapó E., Wojnicki M. (2023). Inorganics.

[cit113] Dejpasand M. T., Saievar-Iranizad E., Bayat A., Montaghemi A., Ardekani S. R. (2020). Mater. Res. Bull..

[cit114] Mondal S., Das S. R., Sahoo L., Dutta S., Gautam U. K. (2022). J. Am. Chem. Soc..

[cit115] Ghaffarkhah A., Hosseini E., Kamkar M., Sehat A. A., Dordanihaghighi S., Allahbakhsh A., van der Kuur C., Arjmand M. (2022). Small.

[cit116] Kaur A., Pandey K., Kaur R., Vashishat N., Kaur M. (2022). Chemosensors.

[cit117] Li Y., Shu H., Wang S., Wang J. (2015). J. Phys. Chem. C.

[cit118] Nesakumar N., Srinivasan S., Alwarappan S. (2022). Microchim. Acta.

[cit119] Kortel M., Mansuriya B. D., Vargas Santana N., Altintas Z. (2020). Micromachines.

[cit120] Zhang H., Guo R., Li S., Liu C., Li H., Zou G., Hu J., Hou H., Ji X. (2022). Nano Energy.

[cit121] Sinha R., Purkayastha P. (2022). Mater. Lett..

[cit122] Kumar N., Yadav S., Sadique M. A., Khan R. (2022). Biosensors.

[cit123] Yan Y., Chen J., Li N., Tian J., Li K., Jiang J., Liu J., Tian Q., Chen P. (2018). ACS Nano.

[cit124] Prabhakar A. K., Ajith M. P., Ananthanarayanan A., Routh P., Mohan B. C., Thamizhchelvan A. M. (2022). OpenNano.

[cit125] Mahalingam S., Manap A., Omar A., Low F. W., Afandi N. F., Chia C. H., Abd Rahim N. (2021). Renewable Sustainable Energy Rev..

[cit126] Felipe B. H. S., Cabral R. L. B., Ladchumananandasivam R., Zille A., Kim S., Fechine P. B. A., Nascimento J. H. O. (2022). J. Mater. Res. Technol..

[cit127] Thomas R., Balachandran M. (2023). J. Cleaner Prod..

[cit128] Yew Y. T., Loo A. H., Sofer Z., Klímová K., Pumera M. (2017). Appl. Mater. Today.

[cit129] Ye R., Xiang C., Lin J., Peng Z., Huang K., Yan Z., Cook N. P., Samuel E. L. G., Hwang C.-C., Ruan G. (2013). Nat. Commun..

[cit130] Mykhailiv O., Zubyk H., Plonska-Brzezinska M. E. (2017). Inorg. Chim. Acta.

[cit131] Bacon R. (2004). J. Appl. Phys..

[cit132] Liu X., Ren J., Licht G., Wang X., Licht S. (2019). Adv. Sustainable Syst..

[cit133] Plonska-Brzezinska M., Butsyk O., Brzezinski K., Sulikowski B., Olejniczak Z., Gras M., Lota G., Molina Ontoria A., Jakubczyk M., Echegoyen L. (2017). Chem.–Eur. J..

[cit134] Mohapatra J., Ananthoju B., Nair V., Mitra A., Bahadur D., V Medhekar N., Aslam M. (2018). Appl. Surf. Sci..

[cit135] Lei J., Liu J., Tang N., Han H., Li Z., Li K., Zhai T., Chen H., Xia H. (2021). Adv. Mater. Interfaces.

[cit136] Dhand V., Yadav M., Kim S. H., Rhee K. Y. (2021). Carbon.

[cit137] Singh V. (2018). Diamond Relat. Mater..

[cit138] Makhongoana A., Matsoso B. J., Mongwe T. H., Coville N. J., Wamwangi D., Maubane-Nkadimeng M. S. (2021). Nanotechnology.

[cit139] Mongwe T. H., Matsoso B. J., Mutuma B. K., Coville N. J., Maubane M. S. (2018). Diamond Relat. Mater..

[cit140] Yeon J. H., Park S. J., Choi I., Choi M. (2019). J. Ind. Eng. Chem..

[cit141] Jin H., Wu S., Li T., Bai Y., Wang X., Zhang H., Xu H., Kong C., Wang H. (2019). Appl. Surf. Sci..

[cit142] Sikeyi L. L., Ntuli T. D., Mongwe T. H., Maxakato N. W., Carleschi E., Doyle B. P., Coville N. J., Maubane-Nkadimeng M. S. (2021). Int. J. Hydrogen Energy.

[cit143] Jiříčková A., Jankovský O., Sofer Z., Sedmidubský D. (2022). Materials.

[cit144] Georgakilas V., Otyepka M., Bourlinos A. B., Chandra V., Kim N., Kemp K. C., Hobza P., Zboril R., Kim K. S. (2012). Chem. Rev..

[cit145] Zhong Y., Mahmud S., He Z., Yang Y., Zhang Z., Guo F., Chen Z., Xiong Z., Zhao Y. (2020). J. Hazard. Mater..

[cit146] Almarzooqi K., Ashrafi M., Kanthan T., Elkamel A., Pope M. A. (2021). Membranes.

[cit147] Cheraghi S., Taher M. A., Karimi-Maleh H., Karimi F., Shabani-Nooshabadi M., Alizadeh M., Al-Othman A., Erk N., Raman P. K. Y., Karaman C. (2022). Chemosphere.

[cit148] Giusto E., Žárská L., Beirne D. F., Rossi A., Bassi G., Ruffini A., Montesi M., Montagner D., Ranc V., Panseri S. (2022). Nanomaterials.

[cit149] Martinez-Martinez R., Islam M. M., Krishnaprasad A., Roy T. (2022). Sci. Rep..

[cit150] Down M. P., Rowley-Neale S. J., Smith G. C., Banks C. E. (2018). ACS Appl. Energy Mater..

[cit151] Güler Ö., Bağcı N. (2020). J. Mater. Res. Technol..

[cit152] Xing X., Zhang X., Zhang K., Jin L., Cao Q. (2019). Fullerenes, Nanotubes Carbon Nanostruct..

[cit153] Sierra U., Álvarez P., Blanco C., Granda M., Santamaría R., Menéndez R. (2016). Fuel.

[cit154] Sierra U., Álvarez P., Blanco C., Granda M., Santamaría R., Menéndez R. (2015). Carbon.

[cit155] Yu H., Guo W., Lu X., Xu H., Yang Q., Tan J., Zhang W. (2021). Food Control.

[cit156] Zhang Q., Sun H., Liu W., Zhou Z., Yuan L., Ren Z., Geng D., Wang J., Cheng X. (2021). Constr. Build. Mater..

[cit157] Sachdeva H. (2020). Green Process. Synth..

[cit158] Fadil Y., Dinh L. N. M., Yap M. O. Y., Kuchel R. P., Yao Y., Omura T., Aregueta-Robles U. A., Song N., Huang S., Jasinski F., Thickett S. C., Minami H., Agarwal V., Zetterlund P. B. (2019). ACS Appl. Mater. Interfaces.

[cit159] Voiry D., Yang J., Kupferberg J., Fullon R., Lee C., Jeong H. Y., Shin H. S., Chhowalla M. (2016). Science.

[cit160] Gu Z., Zhu S., Yan L., Zhao F., Zhao Y. (2019). Adv. Mater..

[cit161] Kumar C., Gupta A., Saharan P., Singh M., Dhakate S. R. (2023). Diamond Relat. Mater..

[cit162] Levchenko I., Baranov O., Riccardi C., Roman H. E., Cvelbar U., Ivanova E. P., Mohandas M., Ščajev P., Malinauskas T., Xu S. (2023). Adv. Mater. Interfaces.

[cit163] Zhan H., Chen Y. W., Shi Q. Q., Zhang Y., Mo R. W., Wang J. N. (2022). Carbon.

[cit164] Holanda E. B. N., Cabral R. L. B., Ladchumananandasivam R., Neto N. F. A., Santos J. E. L., V Santos E., Galvão F. M. F., Bohn F., Nascimento J. H. O. (2022). J. Mater. Sci.: Mater. Electron..

[cit165] Charithra M. M., Manjunatha J. G. (2020). J. Electrochem. Sci. Eng..

[cit166] Sa Y. J., Kim J. H., Joo S. H. (2019). Angew. Chem., Int. Ed..

[cit167] Mao H., Qiu M., Zhang T., Chen X., Da X., Jing W., Fan Y. (2019). Appl. Surf. Sci..

[cit168] Qiu S., Wu K., Gao B., Li L., Jin H., Li Q. (2019). Adv. Mater..

[cit169] Hossain S. M. Z., Mansour N. (2019). Arab J. Basic Appl. Sci..

[cit170] Negri V. , Pacheco-Torres J., Calle D. and López-Larrubia P., Surface-modified Nanobiomaterials for Electrochemical and Biomedicine Applications, 2020, pp. 177–217

[cit171] Wieland L., Li H., Rust C., Chen J., Flavel B. S. (2021). Adv. Energy Mater..

[cit172] Kiran A. R., Kumari G. K., Krishnamurthy P. T. (2020). J. Drug Delivery Sci. Technol..

[cit173] Aslam M. M.-A., Kuo H.-W., Den W., Usman M., Sultan M., Ashraf H. (2021). Sustainability.

[cit174] Kumar R., Singh R. K., Singh D. P. (2016). Renewable Sustainable Energy Rev..

[cit175] Bhattacharjee C. R., Nath A., Purkayastha D. D., Mukherjee B., Sharon M., Sharon M. (2011). Science Journal Ubon Ratchathani University.

[cit176] TermehYousefi A., Bagheri S., Shinji K., Rouhi J., Rusop Mahmood M., Ikeda S. (2014). BioMed Res. Int..

[cit177] Gao L., Li R., Sui X., Li R., Chen C., Chen Q. (2014). Environ. Sci. Technol..

[cit178] Zhuo C., Hall B., Levendis Y., Richter H. (2011). MRS Online Proc. Libr..

[cit179] Lotfy V. F., Fathy N. A., Basta A. H. (2018). J. Environ. Chem. Eng..

[cit180] Karthikeyan S., Mahalingam P. (2010). Int. J. Green Nanotechnol..

[cit181] Xu K., Li Y., Yang F., Yang W., Zhang L., Xu C., Kaneko T., Hatakeyama R. (2014). Carbon.

[cit182] Shao H., Wu Y.-C., Lin Z., Taberna P.-L., Simon P. (2020). Chem. Soc. Rev..

[cit183] Singh G., Lee J. M., Kothandam G., Palanisami T., Al-Muhtaseb A. H., Karakoti A., Yi J., Bolan N., Vinu A. (2021). Bull. Chem. Soc. Jpn..

[cit184] Liu X., Lyu D., Merlet C., Leesmith M. J. A., Hua X., Xu Z., Grey C. P., Forse A. C. (2024). Science.

[cit185] Zhang X., Tian X., Song Y., Wu J., Yang T., Liu Z. (2022). Fuel.

[cit186] Hu X., Radosz M., Cychosz K. A., Thommes M. (2011). Environ. Sci. Technol..

[cit187] Observatory of Economic Complexity , Petroleum Coke Exports, Imports, and Trade Partners, https://oec.world/en/profile/hs/petroleum-coke?yearSelector1=tradeYear1, accessed 6 May 2024

[cit188] Inshakova E., Inshakova A., Goncharov A. (2020). IOP Conf. Ser.: Mater. Sci. Eng..

[cit189] Schmieg E. (1993). Intereconomics.

[cit190] Gargano A., Timmermann A. (2014). Int. J. Forecast..

[cit191] Huang J., Li Y., Zhang H., Chen J. (2021). Int. Rev. Econ. Finance.

[cit192] Robin R. J. (2024). IEEE Eng. Manag. Rev..

[cit193] Geim A. K., Novoselov K. S. (2007). Nat. Mater..

[cit194] U. S. G. Survey , Mineral Commodity Summaries, 2009, Government Printing Office, 2009

[cit195] Zaparolli D. , Grafeno made in Brazil, Pesquisa FAPESP, 2020, vol. 291, p. 1, https://revistapesquisa.fapesp.br/grafeno-made-in-brasil/

[cit196] Xiao Y., Hill J. M. (2020). Chemosphere.

[cit197] Smith C. , City of Chicago Fugitive Dust Study, Chicago, 2014

